# Introduction of organised mammography screening in tyrol: results of a one-year pilot phase

**DOI:** 10.1186/1471-2458-11-91

**Published:** 2011-02-09

**Authors:** Willi Oberaigner, Wolfgang Buchberger, Thomas Frede, Martin Daniaux, Rudolf Knapp, Christian Marth, Uwe Siebert

**Affiliations:** 1 Department of Clinical Epidemiology of the Tyrolean State Hospitals Ltd., Cancer Registry of Tyrol, Innsbruck, Austria; 2 Institute of Public Health, Medical Decision Making and Health Technology Assessment, Department of Public Health, Information Systems and Health Technology Assessment, UMIT - University for Health Sciences, Medical Informatics and Technology, Hall i.T., Austria; 3 ONCOTYROL - Center for Personalized Cancer Medicine, Innsbruck, Austria; 4 Medical Director, TILAK, Innsbruck, Austria; 5 Innsbruck Medical University, Department of Radiology, Innsbruck, Austria; 6 Kufstein County Hospital, Department of Radiology, Kufstein, Austria; 7 Innsbruck Medical University, Department of Obstetrics and Gynecology, Innsbruck, Austria; 8 Center for Health Decision Science, Department of Health Policy and Management, Harvard School of Public Health, Boston, MA, USA; 9 Institute for Technology Assessment and Department of Radiology, Massachusetts General Hospital, Harvard Medical School, Boston, MA, USA

## Abstract

**Background:**

Efficiency and efficacy of organised mammography screening programs have been proven in large randomised trials. But every local implementation of mammography screening has to check whether the well established quality standards are met. Therefore it was the aim of this study to analyse the most common quality indices after introducing organised mammography screening in Tyrol, Austria, in a smooth transition from the existing system of opportunistic screening.

**Methods:**

In June 2007, the system of opportunistic mammography screening in Tyrol was changed to an organised system by introducing a personal invitation system, a training program, a quality assurance program and by setting up a screening database. All procedures are noted in a written protocol. Most EU recommendations for organised mammography screening were followed, except double reading. All women living in Tyrol and covered by social insurance are now invited for a mammography, in age group 40-59 annually and in age group 60-69 biannually. Screening mammography is offered mainly by radiologists in private practice. We report on the results of the first year of piloting organised mammography screening in two counties in Tyrol.

**Results:**

56,432 women were invited. Estimated participation rate was 34.5% at one year of follow-up (and 55.5% at the second year of follow-up); 3.4% of screened women were recalled for further assessment or intermediate screening within six months. Per 1000 mammograms nine biopsies were performed and four breast cancer cases detected (N = 68). Of invasive breast cancer cases 34.4% were ≤ 10 mm in size and 65.6% were node-negative. In total, six interval cancer cases were detected during one year of follow-up; this is 19% of the background incidence rate.

**Conclusions:**

In the Tyrolean breast cancer screening program, a smooth transition from a spontaneous to an organised mammography screening system was achieved in a short time and with minimal additional resources. One year after introduction of the screening program, most of the quality indicators recommended by the European guidelines had been reached.

However, it will be necessary to introduce double reading, to change the rule for BI-RADS 3, and to concentrate on actions toward improving the participation rate.

## Background

Breast cancer is the leading cause of female cancer death in all industrialised countries (and also worldwide), and the breast is also the leading incident cancer site for females [1]. Therefore, screening methods for breast cancer are of greatest public health importance. Efficiency and efficacy of organised mammography screening programs have been proven in large randomised trials conducted in Europe and North America [1-9]. For several years already, organised mammography screening programs have been recommended in the EU [2]. Austria is one of the European countries where up to 2006 no organised programs were implemented, but where coverage by spontaneous mammography screening has been reported to be rather high: in a health survey conducted in Austria in 2006-2007 more than 80% of women aged 40+ answered that they had had at least one mammography (ever) and more than 40% had had one in the past year [10]. However, it is known that self-reporting of screening usage overestimates true coverage [11], and our results are in line with this observation comparing the 40% coverage reported in the above-mentioned health survey to 34.5% in our study. In 2006, the Austrian health minister declared mammography to be one of the top health agendas, and in July 2006 a decision was made to implement organised mammography screening programs, namely in a first step in pilot regions, of which Tyrol is the largest.

Two questions now arise. The first question is whether it is really necessary to change the existing spontaneous mammography system, and severe doubts have been raised [12]. Up to now, our knowledge about the performance of the existing spontaneous program is minimal. We have only very limited information from routine reports from the Cancer Registry of Tyrol giving data on stage distribution of breast cancer cases on the population level and on the time trend of breast cancer incidence and mortality [13]. But it is well agreed that this information is by far not sufficient to assess the quality of a mammography program. To date most quality indices recommended by the EU guidelines cannot be calculated because we do not have the necessary information. It is our opinion that it is not justified to offer a mammography screening system to healthy women without having at least profound information on commonly agreed quality measures.

After that, the second question is how to change the existing screening system in the most efficient manner. It is well accepted that there is no uniform solution for implementing an organised screening system in a country, but, instead, when setting up the mammography system the health system conditions in the respective country must be given consideration. The outstanding challenge in introducing a mammography screening program in a country where a spontaneous screening system was conducted for some time is whether to make a smooth transition to an organised system or to completely redesign the existing screening system as was done, for example, in Germany [14]. In Tyrol, a clear decision was made to set up the new program while making best possible use of the existing experience and mammography screening network, which was established over the last fifteen years. Based on the latter strategy, it was possible to establish a country-wide mammography screening program in very short time. The price to be paid was the risk of potential quality problems, because some EU guidelines concerning the structure of the screening system were not fully adhered to.

To our knowledge, also in Europe a number of countries still have no organised mammography programs [15], and therefore the experience in Tyrol can make an important contribution to the question how to switch a health system with spontaneous mammography screening to an organised system that meets well-accepted quality guidelines.

In June 2007, an organised mammography screening program was introduced in two central counties of Tyrol accounting for forty percent of that state's population. It was the aim of this study to analyse whether a smooth change from a spontaneous to an organised mammography system can meet the quality indicators recommended by the EU guidelines.

## Methods

### Study population, invitation

The target population includes all women aged 40 to 69 living in two counties of Tyrol (i.e., the capital of Innsbruck, and the surrounding area) and covered by compulsory social insurance. More than 97% of the population of Tyrol are covered by compulsory social insurance (personal communication). All women in the target population are sent a personal invitation letter: women aged 40-59 annually, and women aged 60-69 biannually. All women are invited regardless of their screening history and their individual cancer status. Invitations are addressed directly to the women; the invitation is to consult a screening unit. Women invited to screening receive detailed information on the screening programme and must sign an informed consent before screening. Mammography screening is offered by 12 screening units, nine of them run by radiologists in private practice and three by hospital outpatient departments at the two public hospitals in the study area. The mammogram is read by only one radiologist; ultrasound (US) is offered to women at the radiologist's decision. The mammography result is coded according to the BI-RADS [16] scheme, and the participating women are informed of the screening result immediately after the screening test. Women with BI-RADS 1-2 go back to screening, women with BI-RADS 3 are invited for intermediate screening in six months and women with BI-RADS 4 or 5 are invited for further assessment. Assessment is offered by three hospital radiology units in the study area and includes clinical inspection, mammography, US, MRI and biopsy as needed. The one large assessment unit at Innsbruck University Hospital works closely with a breast cancer centre. As the system is open, women are free to contact the assessment unit irrespective of the mammogram result, and a number of the women go to assessment even if the mammography result is BI-RADS 1,2,3. All program activities were planned carefully and documented in a written protocol. The program is directed by a screening group that meets monthly. A subgroup of the project team is responsible for quality assurance, which is based on quarterly analysis of screening data. According to social insurance regulations, women must first visit their general practitioner who refers them to a screening unit. All radiologists participating in the program had to undergo a training program and need ÖRG (Austrian Radiology Association) certification. In the median, private radiologists performed 2450 mammograms and the three hospital units 1134, 1379 and 4620 mammograms per year. There is no waiting time for mammography. Of the women who were referred to assessment, 64% waited less than five working days, 18% between six and ten working days and 19% more than ten working days.

### Data collection

All mammography units register basic information in a database. It is noteworthy that all mammograms are registered, not only those for women belonging to the target population. Due to data privacy restrictions, women must sign a written consent to permit data transfer to the screening database; if a woman refuses consent, an empty dataset marked simply "data transfer declined" is sent to the mammography database. Screening information is transferred to the screening database after pseudonymising the woman's social insurance number. The pseudonymisation process permits linkage of data for a specific woman coming from different units and guarantees data confidentiality, because the pseudonymisation process can be reversed only within the screening unit. An analogous procedure was established for assessment units. Finally, data on tumour characteristics are collected by the Cancer Registry of Tyrol. The Cancer Registry has developed a network of various data sources that guarantees a high degree of completeness and validity of cancer data in the population of Tyrol. Details on registry procedures and figures on completeness have been reported elsewhere [17]. The Cancer Registry also collects each patient's social insurance number and, consequently, Cancer Registry data can be linked to the screening database by applying the same pseudonymisation process. Cancer Registry data enable us to analyse data on tumour characteristics (e.g., tumour size, lymph node status, information on surgery) and to assess interval cancer cases.

Participation rate should reflect all women undergoing a screening mammography. Part of them refuse consent to permit data transfer. In order to account for these women, we estimated the proportion of all women denying consent and belonging to age group 40-69 as being 56% of 5.8%, namely 3.2%. Thus, the estimated participation rate is equal to the observed participation rate plus 3.2%. Finally, invitation data are provided by the invitation system run by the social insurance carrier. Invitation data are transferred to the screening database as aggregated numbers of invited women per month of invitation, age group (five-year age classes) and county. Recall is defined as call for further assessment or invitation to intermediate screening within six months.

### Statistical analysis

Plausibility checks are implemented both at the mammography and the assessment unit level and at the central screening database level.

The screening database is realised as STATA datasets. Linkage between screening data, assessment data and Cancer Registry data is based on the pseudonym number. We report numbers and proportions as defined in the EU guidelines. For some indices, population-based rates are computed using the official population data supplied by Statistics Austria. No statistical testing is applied. All reporting is done with STATA Version 9 [18].

Spontaneous mammography screening was introduced in Tyrol already in the early 1990 s. Thus, the underlying background incidence is defined by years 1988-1990, see Table 1.

**Table 1:** Breast cancer incidence rate in Tyrol before spontaneous screening program 1988-1990 (background incidence) and before changing the screening system 2005-2007

	40-49^1)^	50-59^1)^	60-69^1)^	Total (40-69)^1)^
**1988-1990**				
Breast Cancer	52 (128.8)	54 (176.0)	74 (244.0)	180 (177.5)
Invasive	50 (123.8)	53 (172.7)	72 (238.5)	175 (172.9)
In situ	2 (5.0)	1 (3.3)	2 (5.5)	5 (4.6)
**2005-2007**				
Breast Cancer	82 (146.2)	87 (206.3)	96 (263.0)	265 (196.5)
Invasive	74 (132.0)	79 (187.3)	89 (245.6)	242 (179.9)
In situ	8 (14.2)	8 (19.0)	7 (17.4)	23 (16.6)

^1) ^Average number of incident cases per year and age-specific rate per 100,000

This study was conducted in conformity to the Helsinki Declaration [19]. The project was approved by the Ethics Committee of Medical University Innsbruck.

## Results

From June 2007 to May 2008, 56,432 women in the target population were invited, 40.3% in age group 40-49, 31.1% in age group 50-59 and 28.6% in age group 60-69, see Table 2. A total of 1,188 invitation letters were returned as undeliverable. Because of their small number, we did not take the returned invitations into consideration for analysis. After deleting N = 80 cases without a BI-RADS classification, we ended up with **17,645 screening cases **in the analysis dataset. Of the screening cases 40.4% were in age group 40-49, 32.2% in age group 50-59 and 27.4% in age group 60-69.

**Table 2:** Invitation system: Number of women invited, number of mammograms performed (numbers and age percentages)

	40-49	50-59	60-69	Total (40-69)
Women invited	22739	17531	16162	56432
Number of screens in first year	7124	5689	4832	17645
Observed participation rate in first year	31.3%	32.5%		
Estimated participation rate in first year^1) ^	34.5%	35.7%		
Cumulative participation rate after two years^2)^	54.3%	53.5%	48.2%	52.3%
Estimated cumulative participation rate after two years^1) 2)^	57.5%	56.7%	51.4%	55.5%

^1) ^Also a part of women age 40-59 go to mammography screening at a two year interval.

The observed participation rate in the first year of follow-up was 31.3%; after correcting for women declining data transfer to the screening database, the overall participation rate was 34.5% (34.5%, 35.7% and 33.1% in age groups 40-49, 50-59 and 60-69, respectively). After completing a second year of follow-up (end of observation was 31 May 2009), 55.5% of the invited women had undergone at least one screening examination, with a higher participation rate in younger age classes (57.5%, 56.7% and 51.4% for age groups 40-49, 50-59 and 60-69, respectively), see Table 2.

Screening outcome was negative for 96.6%; 1.6% of cases were recommended for intermediate mammography (six months) after screening (1.2% in age group 60-69), 1.8% were referred for further assessment (1.4% in age group 60-69) and in 10 cases the screening outcome was unknown (Table 3).

**Table 3:** Screening outcome

	40-49	50-59	60-69	Total
Negative	6853 (96.2%)	5482 (96.4%)	4704 (97.4%)	17039 (96.6%)
Intermediate screening test following screening	125 (1.8%)	95 (1.7%)	57 (1.2%)	277 (1.6%)
Assessment				
Recommended	142 (2.0%)	109 (1.9%)	68 (1.4%)	319 (1.8%)
Performed	140 (2.0%)	109 (1.9%)	66 (1.4%)	315 (1.8%)
Unknown^1)^	4	3	3	10
**Total**	**7124**	**5689**	**4832**	**17645**

^1) ^BI-RADS 0 without assessment was treated as unknown

According to screening policy, the screening radiologists were free to perform additional US. The reason for additional US was recorded as breast density grades according to the American College of Radiology (ACR) 3 or 4 in 37.6%, equivocal findings on mammography in 16.9%, and other nonspecified reasons in 45.5% of cases. Overall, 83.3% of women had an additional US examination, with clear differences between the age groups: the proportions were 89.2%, 81.3% and 77.1% in age groups 40-49, 50-59 and 60-69, respectively (Table 4).

**Table 4:** Additional ultrasound imaging at screening

	40-49	50-59	60-69	Total
Ultrasound added to mammography screening	6358 (89.2%)	4624 (81.3%)	3723 (77.1%)	14705 (83.3%)
Reason for ultrasound:				
Breast density (ACR 3/4)	2855 (44.9%)	1638 (35.4%)	1041 (28.0%)	5534 (37.6%)
Equivocal finding	1037 (16.3%)	810 (17.5%)	635 (17.1%)	2482 (16.9%)
Other	2466 (38.8%)	2176 (47.1%)	2047 (55.0%)	6689 (45.5%)

Of 315 assessments performed, 38.4% had a core biopsy (45.5% in age group 60-69), 7.6% a fine needle biopsy and 1.9% (six cases) an open biopsy. This means that of 1000 women screened, nine had a biopsy (8 in age group 60-69) and 0.3 underwent an open biopsy (Table 5). Of all assessments 75.6% were negative, 2.9% were recommended for intermediate screening and in 68 (21.6%) screening cases breast cancer was diagnosed (12.9%, 20.2% and 42.4% in age groups 40-49, 50-59 and 60-69 respectively), see Table 6.

**Table 5:** Assessment procedure

	40-49	50-59	60-69	Total
**Additional imaging**				
Ultrasound	140 (100%)	106 (97.2%)	61 (92.4%)	307 (97.5%)
MRI	24 (17.1%)	21 (19.3%)	21 (31.8%)	66 (21.0%)
**Biopsy**	63 (45.0%)	51 (46.8%)	37 (56.1%)	151 (47.9%)
Core biopsy	51 (36.4%)	40 (36.7%)	30 (45.5%)	121 (38.4%)
Fine needle biopsy	10 (7.1%)	10 (9.2%)	4 (6.1%)	24 (7.6%)
Open biopsy	2 (1.4%)	1 (0.9%)	3 (4.5%)	6 (1.9%)
Biopsy rate per 1000 mammograms	8.8	9.0	7.7	8.6
PPV for core biopsy^1)^	35.3.4%	47.5%	80.0%	50.4%
PPV for open biopsy^2)^				66.7%
**Total**	**140**	**109**	**66**	**315**

^1) ^For all fine needle biopsies the result was benign.

^2) ^For six open biopsies two were benign and four malignant. Because of small numbers, PPV is shown only for the total.

**Table 6:** Assessment outcome

	40-49	50-59	60-69	Total
Negative	117 (83.6%)	85 (78.0%)	36 (54.5%)	238 (75.6%)
Recommendation for intermediate screening after assessment	5 (3.6%)	2 (1.8%)	2 (3.0%)	9 (2.9%)
Breast cancer	18 (12.9%)	22 (20.2%)	28 (42.4%)	68 (21.6%)
In situ	4 (2.9%)	2 (1.8%)	1 (1.5%)	7 (2.2%)
Invasive	14 (10.0%)	20 (18.3%)	27 (40.9%)	61 (19.4%)
Breast cancer detection rate per 1000 mammograms	2.5	3.9	5.8	3.9
Ratio screening breast cancer detection rate vs. background incidence rate^1)^	1.9	2.2	2.4	2.2
**Total**	**140**	**109**	**66**	**315**

^1) ^Background incidence rate: see Table 1

For all fine needle biopsies, the diagnosis was benign. The positive predictive value of core biopsy was 50.4% in total and 35.3%, 47.5% and 80.0% in age groups 40-49, 50-59 and 60-69, respectively. The six open biopsies showed two benign and four malignant cancer cases.

Of the 68 breast cancer cases, 61 were invasive, and seven were ductal carcinoma in situ. The cancer detection rate was 3.9 per 1000 mammograms in total and 2.5, 3.9 and 5.8 in age groups 40-49, 50-59 and 60-69 respectively, see Table 6.

Of all invasive cancers detected, 34.4% were less then 10 mm in size and 65.6% were node-negative, without differences for age group, see Table 7.

**Table 7:** Characteristics of invasive cancers

	40-49	50-59	60-69	Total
Tumour size (mm):	13.5; 3-50	14; 2-60	12; 1-49	13; 1-60
Median; range				
Tumour size (mm):				
<= 10 mm	6 (42.9%)	6 (30.0%)	10 (37.0%)	21 (34.4%)
11-20 mm^1)^	6 (42.9%)	8 (40.0%)	12 (44.4%)	26 (42.6%)
>20 mm	3 (21.4%)	6 (30.0%)	5 (18.5%)	14 (23.0%)
Lymph node involvement	5 (35.7%)	7 (35.0%)	9 (33.3%)	21 (34.4%)
Total	14	20	27	61
Tumor stage according to TNM				
pT1	12 (85.7%)	14 (70.0%)	22 (81.5%)	48 (78.7%)
pT2	2 (14.3%)	5 (25.0%)	5 (18.5%)	12 (19.7%)
pT3		1 (5.0%)		1 (1.6%)
pN0	9 (64.3%)	13 (65.0%)	18 (66.7%)	40 (65.6%)
pN1	4 (28.6%)	4 (20.0%)	6 (22.2%)	14 (23.0%)
pN2	1 (7.1%)	2 (10.0%)	2 (7.4%)	5 (8.2%)
pN3		1 (5.0%)	1 (3.7%)	2 (3.3%)
M0	13 (92.9%)	19 (95.0%)	26 (96.3%)	58 (95.1%)
M1	1 (7.1%)	1 (5.0%)	1 (3.7%)	3 (4.9%)

After linking screening data and Cancer Registry data (only depseudonymised data), we observed a total of six interval cancer cases within one year after screening, four in age group 40-49, one in age group 50-59 and one in age group 60-69. The interval cancer rate was 34 per 100,000 mammograms; this is 19% of the underlying background incidence rate (defined by years of diagnosis 1988-1990). Details are shown in Table 8, which shows the most important quality indicators defined in the EU guidelines restricted to age group 50-69, because EU recommendations are given for that age group.

**Table 8:** EU Guidelines, quality indicators (with accepted and desired levels)

	Tyrol 50-69	EU-accepted	EU-desired
Attendance proportion	54.2	>70	>75
Recall rate^1)^	3.1 (327/10521)	<7%/ <5%	<5%/ <3%
Interval cancer rate/Background incidence rate (BIR) 0-11 months	9% (two interval cancers in age 50-69)	30%	<30%
Breast cancer detection rate	2.3 * BIR	3*BIR	>3*BIR
Stage II+/Total cancers screen-detected	38.0% (19/50)	NA	<30%
Invasive cancers ≤ 10 mm/Total invasive cancers	34.0% (16/47)	NA	>= 25%
Proportion of invasive cancers that are < 15 mm in size	51.1% (24/47)	50%	>50%
Invasive cancer/Total cancers screen-detected	94% (47/50)	90%	80%-90%
Node-negative cancer/Total invasive cancers screen-detected	66.0% (31/47)	NA	>70%
Benign to malignant open surgical biopsy ratio	0:1 (0:4)	<=1:2	<=1:4
Benign to malignant core biopsy ratio	1:0.9 (60:64)		

^1) ^Recall is defined as call for further assessment or invitation to intermediate screening within six months

## Discussion

We analysed the situation after a first year of introducing organised mammography screening in two counties in Tyrol accounting for 40% of that state's population. The organised program was established in a smooth transition from the existing spontaneous mammography screening system, namely by introducing a written protocol, a personal invitation system, a training program, and by setting up a screening database allowing us to investigate performance and outcome parameters in detail. Although not all EU recommendations have been followed to date, most quality indicators are in the range of accepted and/or desired levels given by the EU guidelines: the proportion of cases called for further assessment (20 per 1000 mammograms), the biopsy rate (9 per 1000 mammograms), the proportion of invasive screening-detected cancer (89.7%), the proportion of invasive cancer ≤ 10 mm in size (34.4%) and the interval cancer rate expressed as a multiple of the background incidence rate (19%). The average number of screens read by a radiologist (about 2400) does not meet the EU recommendation of 5000. However, in about four of five women an additional US is done, which is known to improve the sensitivity of the screening test, see for example [20,21].

Some of the parameters are near the value expected in subsequent screening rounds and not in a first round (for example, recall rate and breast cancer detection rate). However, we must remember that the organised program was introduced after fifteen years of the spontaneous screening program. Some indicators like proportion of stage II+ cancers and node-negative cancer are slightly outside the EU-accepted levels. Some of the observations could be due to small numbers (we observed a total of 68 cancer cases). So it is too early to come to final conclusions on the mammography screening model we describe here.

Only one indicator clearly misses the EU recommendations, namely the participation rate. However, participation rate depends not only on program organisation, but also on cultural background. A look at neighbouring German-speaking countries, which should have a similar culture, shows participation rates of 54% in Germany [14] and 25.9% to 65.9% in five cantons of Switzerland [22]. Thus, a cumulative participation rate of 55% in the first two years would seem to be rather successful by comparison to that of countries with a similar cultural background, albeit not the goal we aimed for.

Among the strengths of the Tyrolean breast cancer screening program is its implementation: within a short time and with minimal additional resources it was possible to set up an organised population-based screening program that - at least at evaluation after one year - met all of the EU quality indicators except participation rate. It was not necessary to set up extra screening units; instead, the program used the existing network of screening and assessment units. In terms of epidemiology, another of the program's strengths is a complete pseudonymised database that covers all mammography exams and can be linked to the Cancer Registry data. The database was set up in a very short time, it is up to date within one month and serves as a very important tool for monitoring the program quality indicators or, more generally speaking, for all kinds of quality assurance tasks.

Nevertheless, the program differs from many organised programs in the EU in three aspects. Firstly, we also included women aged 40-49. During previous spontaneous screening campaigns women aged 40-49 were officially invited to participate in mammography screening. The discussion in the USA after publishing the revised recommendation by USPSTF [23,24] shows that it is very challenging and difficult for women to understand why a service is cancelled that was recommended for several years. Without well founded data, we feel it is not justified to discontinue screening in age class 40-49. We are collecting detailed data and will evaluate the goods and harms [23,25] in coming years. In addition, the analysis of breast cancer mortality in Tyrol in the past decade shows that mortality decrease was greatest in women aged 40-49, an effect that can at least partly be attributed to spontaneous screening [13].

Secondly, we offer breast US as an additional diagnostic tool in screening. In opportunistic screening in Tyrol, US was offered to women with dense breasts (ACR density grades 3 and 4) and with inconclusive findings on mammography [26,27]. The line of reasoning concerning screening in age group 40-49 also holds for adjunct US, bearing in mind that adjunct US was offered during the last decade. It has been shown by various authors that the additional use of US can improve cancer detection rates, especially in younger women and women with dense breasts [20,21,26]. The relative percentage of carcinomas found in supplemental breast US examinations as a fraction of the total number of detected cancers was reported by four studies with a mean percentage of 22.5% (15%-34%) [19]. In the second year of the organised program we collected detailed data allowing us to analyse the contribution of US to sensitivity and specificity outside the framework of studies, namely in a population-based setting.

And thirdly, we were not able to implement double reading during the piloting phase. Interestingly, performance parameters, especially interval cancer rate, showed that also without double reading an acceptable quality level was achieved. The question of the completeness of interval cancer rate depends on the completeness of the Cancer Registry of Tyrol, which covers the target population. We analysed quality measures for the Cancer Registry in detail, also completeness, and found a high degree of completeness, both for all cancer sites and for breast cancer [17]. Therefore, we think it is unlikely that we missed interval cancers. However, the numbers are small and we cannot exclude the possibility that we missed one or two cases, which would mean a 33% increase in the interval cancer rate (from 6 to 8 cases). Clearly, a longer observation period is necessary before coming to a final conclusion on this parameter. This observation could in part be explained by short screening intervals in the age group 40-59 and by the use of additional US. Nevertheless, double reading will be introduced in the regular screening program after concluding the piloting phase.

Also, as we do not have a scheduling system, women are invited to consult the screening unit at any time that is convenient to them. The time when the invitation is sent is independent of a woman's individual screening history. The latter point is corrected in the next invitation round, which required a change in the written consent for reasons of data privacy.

Many countries have had a mammography screening program running for decades or for a shorter time. On the other hand, six EU member states still have no organised nationwide breast cancer screening program, and in seven member states a nationwide rollout was in 2007 [15]. What can be learned from our experience by countries that are thinking of introducing or are already in the process of planning to introduce a mammography screening program? In our opinion, the greatest difference between our approach and many other approaches is the smooth transition made from the existing spontaneous program to organized population-based screening. We made use of the network of screening and assessment units that had already been set up during spontaneous screening. What we added was an invitation system covering the entire population of Tyrol, a screening database that allows quality indices to be monitored and a well-defined training program for both screening and assessment units. With this strategy we were able to meet most EU quality indices in a very short time.

## Conclusions

A smooth transition from a spontaneous to an organised mammography screening system that uses the existing screening units, introduces an invitation system and a quality assurance program (including a screening database) can meet the quality indicators recommended by European guidelines in a short time and with minimal additional resources. However, it will be necessary to introduce double reading, to change the rule for BI-RADS 3 and to concentrate on actions toward improving the participation rate.

## List of Abbreviations

US: ultrasound; ÖRG: Austrian Radiology Association; ACR: American College of Radiology.

## Competing interests

The authors declare that they have no competing interests.

## Authors' contributions

WO designed the study, performed the analysis and wrote the paper. WB contributed to study design and assisted in writing the paper, especially contributions on ultrasound and screening at age below 50. TF, MD und RK contributed to the discussion part, especially from the radiology point of view. CM contributed to the methods, report and discussion part, especially from the gynaecological point of view. US contributed to writing of the paper. All Authors reviewed and agreed to the final version of the manuscript.

## Pre-publication history

The pre-publication history for this paper can be accessed here:

http://www.biomedcentral.com/1471-2458/11/91/prepub
